# Levels of Anti-Citrullinated Protein Antibodies and Rheumatoid Factor, Including IgA Isotypes, and Articular Manifestations in Ulcerative Colitis and Crohn’s Disease

**DOI:** 10.3390/ijerph17218054

**Published:** 2020-11-01

**Authors:** Koen M. J. Janssen, Hilde Hop, Arjan Vissink, Gerard Dijkstra, Menke J. de Smit, Elisabeth Brouwer, Johanna Westra

**Affiliations:** 1 Department of Oral and Maxillofacial Surgery, University of Groningen and University Medical Center Groningen, P.O. Box 30.001, 9700 RB Groningen, The Netherlands; kmj.janssen@gmail.com (K.M.J.J.); m.j.de.smit@umcg.nl (M.J.d.S.); 2 Department of Internal Medicine, University of Groningen and University Medical Center Groningen, P.O. Box 30.001, 9700 RB Groningen, The Netherlands; h.hop@umcg.nl; 3 Department of Gastroenterology and Hepatology, University of Groningen and University Medical Center Groningen, P.O. Box 30.001, 9700 RB Groningen, The Netherlands; gerard.dijkstra@umcg.nl; 4 Department of Rheumatology and Clinical Immunology, University of Groningen and University Medical Center Groningen, P.O. Box 30.001, 9700 RB Groningen, The Netherlands; e.brouwer@umcg.nl (E.B.); johanna.westra@umcg.nl (J.W.)

**Keywords:** rheumatoid arthritis, inflammatory bowel disease, ulcerative colitis, Crohn’s disease, anti-citrullinated protein antibodies, arthralgia

## Abstract

Systemic presence of arthritis autoantibodies (AAb) is specific for rheumatoid arthritis (RA). AAb initiation might be triggered by chronic mucosal inflammation, such as in inflammatory bowel disease (IBD). We assessed the prevalence of anti-citrullinated protein antibodies (ACPA) and rheumatoid factor (RF) in ulcerative colitis (UC) and Crohn’s disease (CD) patients, with regard to the prevalence of joint complaints in AAb+ versus AAb− IBD patients. RA patients and healthy subjects (HC) served as controls. Serum was collected from 226 UC, 165 CD and 86 RA patients, and 36 HCs. One-hundred-and-ten UC (48.7%) and 76 CD (46.1%) patients were seropositive for at least one autoantibody, compared to 4 (13.9%) HCs and 81 (94.2%) RA patients. Eighty-three (37%) UC and 52 (32%) CD patients were seropositive for the anti-cyclic citrullinated protein antibody (anti-CCP2) of the immunoglobulin A type (IgA anti-CCP2), compared to 1 (2.8%) HC and 64 (74%) RA patients. RF of the immunoglobulin G type (IgG RF) and IgA RF seropositivity in UC and CD patients was comparable to HCs and low compared to RA patients. Arthralgia was reported by 34 (18.7%) UC and 50 (33.1%) CD patients, but presence of arthralgia was not increased in AAb+ patients. AAbs are frequently present in IBD patients, supporting the hypothesis that inflammation of intestinal mucosa induces low systemic levels of ACPA.

## 1. Introduction

Inflammatory bowel disease (IBD) is characterized by inflammation of the intestinal mucosa. Ulcerative colitis (UC) and Crohn’s disease (CD) are the main subtypes. UC and CD differ in the location and nature of the disease [1,2]. UC is usually characterized by involvement of the colon with acute and chronic inflammation limited to the mucosa, while inflammation can be transmural in any part of the gastrointestinal tract in CD patients. Extra-intestinal manifestations are often present in UC and CD patients, of which peripheral arthropathy is most commonly observed [2,3,4].

Certain antibodies have been described to be present in UC and CD patients. UC is associated with the presence of perinuclear anti-neutrophil cytoplasmic antibodies (pANCA) [5], while CD is characterized by antibodies against microbes such as *Saccharomyces cerevisiae* (ASCA). Both antibodies were found to have a high specificity but a rather low sensitivity for UC and CD, which hampers their clinical utility. However, the presence of these autoantibodies is associated with a more severe disease phenotype in UC and CD [6]. These antibodies can already be present in serum years before the diagnosis of UC or CD is made [7].

An important hallmark for rheumatoid arthritis (RA) is the presence of rheumatoid factor (RF) and anti-citrullinated protein antibodies (ACPA), the latter being the most specific for RA [8,9]. The role of these autoantibodies in RA pathogenesis is currently unclear, but their presence in RA patients is associated with a more severe disease outcome [10]. Furthermore, RA is often preceded by the presence of these autoantibodies years before the clinical onset [11]. 

During inflammation, when apoptosis and necrosis take place, citrullination of proteins occurs in the intestinal tissue of IBD patients [12,13]. Citrullinated proteins are generated via the conversion of arginine into citrulline by a posttranslational process mediated by the enzyme peptidyl arginine deiminase (PAD). Citrullinated epitopes are created during this process, which might be targeted by ACPA, a phenomenon that also occurs in RA. Therefore, the initiation of ACPA production has been hypothesized to take place at inflamed mucosal surfaces in predisposed individuals, e.g., in the gastrointestinal tract [14,15], via PAD release by neutrophils, or exposure to citrullinated proteins by neutrophil extracellular traps (NETs) [16]. This hypothesis is supported by the capability of plasma cells in inducible bronchus-associated lymphoid tissue from RA patients to produce ACPA locally [17].

In the current study, we measured the serological levels of ACPA (of the immunoglobulin A (IgA) and immunoglobulin G (IgG) type) and RF (IgM and IgA) in a cross-sectional group of IBD patients. It is reported in the literature that IgA ACPA [18,19] and IgG ACPA [20,21,22] have a low prevalence in IBD patients. None of these studies assessed the presence of low ACPA levels below the diagnostic cut-off, however. Diagnostic cut-off means the level that is used for diagnosis of the RA. For the IgG anti-cyclic citrullinated protein antibody (anti-CCP2), this level is 25 U/mL when using the commercial anti-CCP2 ELISA (Enzyme-Linked Immuno Sorbent Assay). We presume that a rise of ACPA levels compared to healthy controls, even below diagnostic cut off, might indicate the initiation of ACPA production. Therefore, the objective of this study was to assess whether IgA ACPA, IgG ACPA, IgA RF and IgG RF were present in IBD patients (UC and CD) as well as to assess the prevalence of arthropathies, especially the presence of arthralgia, in autoantibody positive and autoantibody negative IBD patients. The combination of the presence of ACPA and/or RF and arthralgia has been shown to increase the risk for future RA development [15,23].

## 2. Materials and Methods 

### 2.1. Patients

A total of 391 IBD patients (226 patients with UC and 165 with CD) participating in the Dutch IBD Pearl Chain Institute Biobank Initiative were included. All patients gave their informed consent before study enrollment. Patients were included when there was a confirmed diagnosis for UC or CD. Patients were examined by a gastroenterologist at the outpatient clinic of the University Medical Center Groningen, who asked about the presence of arthralgia. The diagnosis of arthritis and spondylarthropathies needed to be confirmed by a rheumatologist. Blood was withdrawn at study enrollment and serum was stored at −80 °C until further use. Clinical and demographic information, including age, gender, diagnosis, duration of disease, body mass index (BMI), and smoking habits were standardized and recorded at the time of blood withdrawal. Serological parameters, such as pANCAs, anti-ASCA (IgA and IgG), C-Reactive Protein (CRP) levels and erythrocyte sedimentation rate (ESR) were routinely measured. CRP was measured by turbidimetry with a lower limit of detection ≤3 mg/L.

An HC group (n = 36), consisting of persons without systemic disease [24], was included to define cut off-levels for autoantibody levels. Additionally, 86 RA patients were included as a reference group for serological measurements. The study was conducted with the approval of the Medical Ethics Committee of the University Medical Center Groningen.

### 2.2. Laboratory Measurements

Anti-cyclic citrullinated protein antibody (anti-CCP2) levels were measured using the commercial anti-CCP2 ELISA (Eurodiagnostica). The manufacturer’s protocol was adjusted to measure specifically low IgG anti-CCP2 levels as earlier described [16]. Samples were diluted 1:10 instead of 1:50 in dilution buffer. Seropositivity was defined as >2SD above the mean of HC (2.2 U/mL). 

The specific ACPA response of the 20 IBD patients with the highest IgG anti-CCP2 levels was measured with ELISA by testing the reactivity against 4 well-known citrullinated antigens, which are often recognized by ACPA from RA patients. These well-known citrullinated antigens were two peptides from fibrinogen (Fib1, β-chain amino acids 36–52, NEEGFFSACitGHRPLDKK and Fib2, β-chain amino acids 60–74, CitPAPPPISGGGYCitACit), one peptide from α-enolase (Eno1, KIHACitEIFDSCitGNPTVE), and one peptide from vimentin (Vim1, VYATCitSSAVCitLCitSSV). Citrulline-specific IgG reactivity was determined by measuring the difference in reactivity against the citrullinated and native form of the peptides, with the cut-off defined as the difference in optical density (ΔOD) >2SD above the mean of HCs, as previously described [24].

IgA anti-CCP2 levels were measured using a modified anti-CCP2 assay. Sera were diluted 1:50 using the dilution buffer provided by the manufacturer. Bound human IgA was detected by the secondary antibody horseradish peroxidase (HRP)-conjugated polyclonal goat anti-human IgA (SouthernBiotech, Birmingham, AL, USA), diluted in phosphate-buffered saline (PBS) with 1% bovine serum albumin (BSA) and 0.05 % Tween-20 (Sigma-Aldrich, St. Louis, MO, USA). The color reaction was performed using tetramethylbenzidine (Sigma-Aldrich) and hydrogen peroxide. A pool of sera from 4 RA-patients with high IgA anti-CCP2 levels served as a calibrator for the standard curve expressed in arbitrary units per milliliter (AU/mL) and starting at 200 AU/mL. Seropositivity was defined as >2 SD above the mean of HCs.

IgM and IgA RF levels in serum were measured by a validated in-house ELISA assay [25]. Levels were expressed in international units per milliliter (IU/mL), and seropositivity was defined as >10 IU/mL for IgM RF and >25 IU/mL for IgA RF.

### 2.3. Statistical Analysis

Statistical analysis was performed with GraphPad Prism software (version 5.00 for Windows, GraphPad Software, La Jolla, CA, USA) and IBM SPSS Statistics for Windows software (version 20.0, IBM, Armonk, NY, USA). For group comparisons, a Mann–Whitney U test was used for continuous variables, and Fisher’s exact test or Chi-square test for categorical variables. Values of *p* < 0.05 were considered to be significant.

## 3. Results

### 3.1. General Characteristics

The demographic and clinical characteristics of patients and healthy controls are depicted in Table 1. UC patients had a significantly higher BMI than CD patients (*p* = 0.001), while the CD-cohort was comprised of more (ever) smokers than the UC cohort (*p* = 0.009). The disease duration was similar in both groups. 

### 3.2. Autoantibody Seropositivity

Overall, IgG anti-CCP2 levels were low when the diagnostic value in RA diagnosis was taken into account (25 U/mL). In contrast to the RA patients, none of the IBD patients displayed levels above the diagnostic cut-off value of RA, with the highest level being 13.6 U/mL in the IBD patients. However, when seropositivity was defined using a lower cut-off, >2SD above the mean of HCs, 29 UC patients (13%) and 28 CD patients (17%) were seropositive for IgG anti-CCP2, while 2 (5.6%) HCs and 74 (86%) RA patients were seropositive. Additionally, the specific IgG response against four citrullinated peptides (Fib1, Fib2, Eno1 and Vim1) was measured in 20 sera from IBD patients with the highest IgG anti-CCP2 response, all being higher than 4 U/mL. None of these IBD patients showed citrulline-specific reactivity against any of these four peptides. Therefore, citrulline-specific reactivities against these peptides were not measured in any of the other IBD patients, as no positive results were to be expected.

IgA anti-CCP2 levels are generally not used in RA diagnosis, therefore no diagnostic cut-off is known. When applying a similar cut-off, as was used for IgG anti-CCP2, >2SD above the mean of HCs, 83 UC patients (37%) and 52 CD patients (32%) were seropositive for IgA anti-CCP2, while only 1 (2.8%) HC and 64 (74%) RA patients were.

IgM and IgA RF seropositivity was low with 10 (4.4%) patients being seropositive for IgM RF, 6 (2.7%) for IgA RF in UC patients, 6 (3.6%) for IgM RF, and 8 (4.8%) in CD patients, respectively, compared to 1 the (2.8%) in HCs for both IgM RF and IgA RF. 64 (74%) and 43 (50%) RA patients were seropositive for IgM RF and IgA RF, respectively.

**Figure 1:**
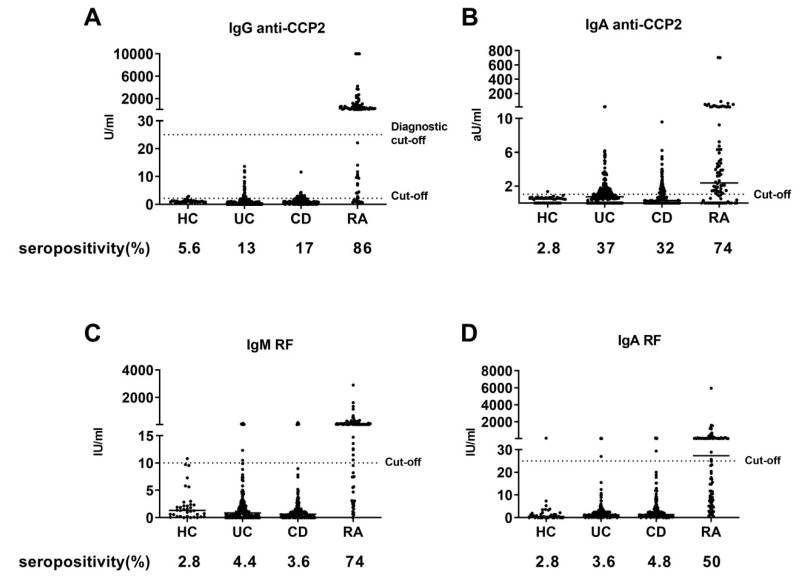
Arthritis Autoantibody (AAb) levels for healthy controls (HC, n = 36), Ulcerative colitis (UC, n = 226), Crohn’s disease (CD, n = 165) and rheumatoid arthritis (RA, n = 86) patients. (**A**) IgG anti-CCP2 levels; (**B**) IgA anti-CCP2 levels; (**C**) IgM RF levels and (**D**) IgA RF levels. U/mL = Units/mL; aU/mL = arbitrary units/mL; IU/mL = International units/mL.

**Figure 2:**
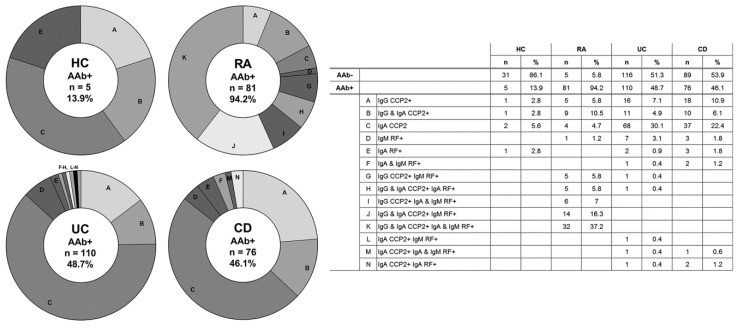
Distribution of arthritis autoantibody (AAb) positivity in HC (n = 5), RA (n = 81), UC (n = 110) and CD (n = 76) patients, represented in pie charts.

In total, 110 UC patients (48.7%) and 76 CD patients (46.1%) were seropositive for at least one arthritis autoantibody, of which the majority were positive for IgA anti-CCP2. The autoantibody levels are shown in Figure 1, while seropositivity in the groups is depicted in Figure 2.

### 3.3. Rheumatic Manifestations and Arthritis Autoantibody Status 

Next, IBD patients who were seropositive for any of the arthritis autoantibodies (AAb+) were compared to arthritis autoantibody negative (AAb−) IBD patients on clinical manifestations (Table 2). Over 90% of AAb+ patients were seropositive for IgA and/or IgG anti-CCP2. AAb+ and AAb− patients did not differ in smoking behavior, disease duration or disease activity. AAb+ positive patients were significantly younger than AAb− patients (UC *p* = 0.016, CD *p* = 0.017). AAb+ UC patients were significantly younger at the disease onset than AAb− UC patients (*p* = 0.021). CRP levels higher than 3 mg/L were seen in 21% of AAb− UC patients and 22% of AAb+ UC patients, while this level was 18% in AAb− CD patients and 38% in AAb+ CD patients. ESR (*p* = 0.009) and CRP (*p* = 0.008) levels were significantly increased in AAb+ CD patients compared to AAb− CD patients.

The presence of arthralgia was reported in 34 (18.7%) UC-patients and 50 (33.1%) CD-patients. Remarkably, in AAb+ IBD patients, the presence of arthralgia was not increased. Articular manifestations other than arthralgia were reported in few patients not allowing for any statistical analysis. Treatment in UC and CD consisted of (a combination of) immunosuppressive drugs, mesalazine, steroids, azathioprine, methotrexate, and anti-Tumor Necrosis Factor (TNF) agents. Anti-CCP2 and/or RF seropositivity was not associated with any of these therapies.

## 4. Discussion

Inflamed mucosal surfaces (e.g., intestinal tissue, lungs and periodontium) have been hypothesized as sites where the formation of ACPA initiates. The lungs [17,19] and periodontium [26,27] have already been studied as potential sites for AAb generation, while the intestinal area is still to be assessed as a site where autoantibody generation starts. The current study revealed that AAbs, especially ACPA of both IgA and IgG subclasses, are present in serum from IBD patients. ACPAs, especially of the IgA subclass, are increased in IBD patients compared to HCs, but do not exceed the diagnostic level for RA [24]. IgM RF and IgA RF seropositivity was comparable to that observed in the general population [28]. With regard to IBD patients with arthralgia, AAb+ IBD patients were significantly younger; the implications of this observation is not straightforward and was unexpected, as AAb positivity is more prevalent among the aged [29]. The higher ESR and CRP levels in AAb+ than in AAb− CD patients confirm the hypothesis that low positive ACPA levels are associated with inflammation. Although CD and UC are both inflammatory diseases, CRP is a less reliable marker of inflammation and disease activity in patients with UC, perhaps except for severe, extensive colitis [30].

Environmental and genetic factors are thought to play a role in the development of ACPA. Environmental factors such as smoking were shown as risk factors for developing ACPA-positive RA [15,31,32]. Smoking is considered to induce ACPA via the citrullination of proteins in the lungs by activating the enzyme peptidyl arginine deiminase (PAD). Our results showed that the AAb− and AAb+ IBD patients did not differ in smoking behavior. The presence of citrullinated proteins in colonic biopsies from IBD patients [12,33] might be on the basis of the higher systemic ACPA levels in IBD patients, similarly as smoking is able to induce citrullination in the lungs [34]. 

Genetic factors for ACPA development in RA, especially human leukocyte antigen DR beta 1 (HLA-DRB1) shared epitope (SE) alleles, are thought to play a role in the epitope spreading and rise of ACPA levels after the initiation of ACPA production, which is HLA-DRB1 SE independent [35]. In addition, bacterial antigens are hypothesized to contribute to ACPA development via molecular mimicry of self-antigens presented by predisposing HLA-DQ molecules [36]. However, HLA-associated genetic risk factors involved in IBD [37,38] are not involved in development of ACPA+ RA [39]. Therefore, we can speculate that due to the absence of these HLA-related risk factors, ACPA initiation in IBD is not followed by a maturation of the ACPA response and spreading of epitopes, which can be measured in the context of RA development. This is also reflected by the negative results on the reactivity against the four citrullinated peptides, although this assay is also less sensitive than the anti-CCP2 ELISA.

The results of our study show that extra-intestinal manifestations in IBD patients are not associated with arthritis autoantibody levels in serum. The presence of arthralgia was reported in a high number of patients (34 (18.7%) UC patients and 50 (33.1%) CD patients). Arthralgia was not clearly defined; therefore, it could have been that enthesitis and fibromyalgia were also in part responsible for these large numbers. Notwithstanding, our study confirms the results of other studies that found no association between articular manifestations and ACPA in IBD patients [21,22,40]. 

IBD is a condition which is particularly linked to gut microbiota, especially to gammaproteobacteria and the presence of *Escherichia coli* and fusobacteria [41,42,43,44]. The link between RA and the human microbiome is less clear [45]. The oral anaerobe bacterium *Porphyromonas gingivalis* has been hypothesized to play a role in RA development by creating citrullinated bacterial and/or human antigens with its own PAD-enzyme (PPAD) [46]. However, oral microbiome studies [47,48] did not find associations with *P. gingivalis* in the oral microbiome and the presence of RA. Thus, enzymatic activity of PPAD is probably not a major virulence mechanism during the early stages of inflammatory arthritis [49]. Studies on gut-microbiota in treatment-naive RA patients point towards an increased prevalence of *Prevotella copri* in early RA patients [50] as well as *Lactobacillus salivarius* [48]. Zhang et al [48] studied the relationship between the gut microbiome and autoantibody levels and found that *Haemophilus* spp. were negatively correlated with anti-CCP, while the presence of *Prevotella* spp. positively correlated with RF. Whether gut-microbiota also play a role in articular manifestations and autoantibody levels in IBD patients remains a topic for future research. 

A limitation of the current study was that not all UC and CD patients were seen by a rheumatologist. Only in case of a suspect of arthritis and spondylarthropathies was the patient seen by a rheumatologist. Another limitation of the study was that we could not relate arthritis autoantibodies to gut microbiome data, which would have been very interesting, since such an association has been suggested in several studies.

## 5. Conclusions

In conclusion, arthritis autoantibodies are present in serum from IBD patients, which supports the hypothesis that the inflammation of intestinal mucosa induces low systemic levels of ACPA, although their role in the induction of RA is yet unclear. The development of arthralgia in IBD patients is independent of the presence of arthritis autoantibodies.

## Figures and Tables

**Table 1:** Subject characteristics.

Characteristics	Healthy Controls (HC) (n = 36)	Rheumatoid Arthritis (RA) Patients (n = 86)	Ulcerative Colitis (UC) Patients (n = 226)	Crohn’s Disease (CD) Patients (n = 165)	UC vs. CD *p* Value
**Female, n (%)**	20 (55.6)	60 (70)	123 (54.4)	105 (63.6)	0.077
**Age, years, mean, (SD)**	34 (15)	55 (11)	42 (15)	41 (15)	0.606
**BMI, kg/m^2^, mean, (SD) ^a^**	–	–	26.1 (4.6)	24.8 (4.9)	0.001
**Current or ever smoker, n (%) ^b^**	8 (22.3)	34 (39)	129 (57.1)	108 (67.1)	0.006
**DAS28, median, IQR**	–	2.2 (1.7–2.8)	–	–	–
**Disease duration, years, (median, IQR)**	–	5.5 (3–10)	7 (4–13)	9 (4–16)	0.037
**Montréal classification**					
**Age at diagnosis, n (%) ^c^**					0.092
**A1 below 16 years**	–	–	20 (9.0)	26 (16.2)	
**A2 between 17 and 40 years**	–	–	146 (65.8)	101 (62.7)	
**A3 above 40 years**	–	–	56 (25.2)	34 (21.1)	
**Disease extent (E) and severity**					
**(S) in UC, n (%) ^d^**					
**E1 ulcerative proctitis**	–	–	37 (17.0)	–	–
**E2 left sided UC**	–	–	74 (33.9)	–	–
**E3 extensive UC**	–	–	107 (49.1)	–	–
**S0 clinical remission**	–	–	20 (9.3)	–	–
**S1 mild UC**	–	–	68 (31.8)	–	–
**S2 moderate UC**	–	–	72 (33.6)	–	–
**S3 severe UC**	–	–	54 (25.2)	–	–
**Disease location (L) and**					
**behavior (B) in CD, n (%) ^e^**					
**L1 ileal**	–	–	–	57 (36.3)	–
**L2 colonic**	–	–	–	32 (20.4)	–
**L3 ileocolonic**	–	–	–	67 (42.7)	–
**L4 isolated upper disease**	–	–	–	1 (0.6)	–
**B1 non-stricturing, non-penetrating**	–	–	–	83 (51.2)	–
**B2 stricturing**	–	–	–	56 (34.6)	–
**B3 penetrating**	–	–	–	23 (14.2)	–
**P perianal disease modifier**	–	–	–	45 (27.8)	–

^a^ 225/226 UC and 164/166 CD; ^b^ 161/165 CD; ^c^ Age known for 222/226 UC and 161/165 CD; ^d^ According to Montréal classification, A (age at diagnosis), E (Extent) known for 218/226 and S (Severity) known for 214/226 UC; ^e^ L (Location) known for 157/165 CD and B (Behavior) known for 162/165 CD. Analysis: Mann–Whitney U test for continuous variables and Fisher exact or Chi-Square test for categorical variables. BMI, body mass index; DAS28, Disease Activity Score 28, tender and swollen joint count.

**Table 2:** Comparison between serological characteristics and articular manifestations in inflammatory bowel disease (IBD) patients subdivided for seropositivity for at least one rheumatoid arthritis (RA) related autoantibody.

	Ulcerative Colitis			Crohn’s Disease		
Characteristics and manifestations	AAb− (n = 116)	AAb+ (n = 110)	*p* Value	AAb− (n = 89)	AAb+ (n = 76)	*p* Value
**Female, n (%)**	67 (57.8)	56 (50.9)	0.35	55 (61.8)	50 (65.8)	0.624
**Age, years, mean (SD)**	44.1 (15)	39.4 (14)	0.016	43.6 (15)	38.0 (14)	0.017
**Ever smoker, n (%)**	70 (60.3)	59 (53.6)	0.348	60 (69.0)	48 (64.9)	0.616
**Active disease, n (%) ^a^**	34 (29.3)	35 (31.8)	0.661	39 (47.6)	29 (42.7)	0.622
**Age at diagnosis, n (%)**			0.021			0.159
**A1 below 16 years**	9 (7.8)	11 (10.3)		10 (11.2)	16 (22.2)	
**A2 between 17 and 40 years**	68 (59.1)	78 (72.9)		60 (67.4)	41 (56.9)	
**A3 above 40 years**	38 (33.0)	18 (16.8)		19 (21.3)	15 (20.8)	
**Disease duration, years, median (IQR)**	7 (4–14)	7 (4–12)	0.425	11 (4–17)	8.5 (4–15)	0.264
**ESR, mm/h, median (IQR)**	11 (5–18)	11 (5–18)	0.974	9 (5–20)	17.5 (8–24)	0.009
**CRP, mg/l, median (IQR)**	≤3 (≤3–3)	≤3 (≤3–3)	0.865	≤3 (≤3–3)	≤3 (≤3–8)	0.008
**pANCA, seropositive (%) ^b^**	51 (52.7)	54 (61.4)	0.239	20 (25.6)	18 (29.5)	0.702
**ASCA, seropositive (%) ^c^**	24 (25.8)	24 (27.3)	0.867	48 (61.5)	40 (69.0)	0.582
**Arthropathies**						
**Enthesitis, n**	0	1	-	3	0	-
**Arthritis, n**	0	0	-	0	1	-
**Inflammatory backpain, n**	0	0	-	3	0	-
**Arthralgia, n (%) ^d^**	13 (14.7)	21 (22.3)	0.254	26 (32.5)	24 (34.3)	0.863

AAb−, arthritis autoantibody negative; AAb+, arthritis autoantibody positive; ^a^ measured by simple clinical colitis activity index (SCCA) ≥3 or Harvey-Bradshaw index ≥4; ^b^ known for 180/226 UC and 139/165 CD; ^c^ Defined as IgA and/or IgG ASCA seropositive, known for 181/226 UC and 136/165. ^d^ known for 182/226 UC and 151/165 CD; A, age at diagnosis; ESR, erythrocyte sedimentation rate; CRP, C-reactive protein; pANCA, Perinuclear Anti-Neutrophil Cytoplasmic Antibodies; ASCA, anti-*Saccharomyces cerevisiae* antibodies.
